# Multi-layer matrix factorization for cancer subtyping using full and partial multi-omics dataset

**DOI:** 10.1093/bib/bbaf448

**Published:** 2025-09-09

**Authors:** Yingxuan Ren, Fengtao Ren, Bo Yang

**Affiliations:** National University of Singapore, 119077, Singapore; Department of Engineering, The Chinese University of Hong Kong, 999077, Hong Kong, China; School of Computer Science, Xi'an Polytechnic University, 710048, Xi'an, China

**Keywords:** matrix factorization, cancer subtyping, missing data, multi-omics data

## Abstract

Cancer, with its inherent heterogeneity, is commonly categorized into distinct subtypes based on unique traits, cellular origins, and molecular markers specific to each type. However, current studies primarily rely on complete multi-omics datasets for predicting cancer subtypes, often overlooking predictive performance in cases where some omics data may be missing and neglecting implicit relationships across multiple layers of omics data integration. This paper introduces Multi-Layer Matrix Factorization (MLMF), a novel approach for cancer subtyping that employs multi-omics data clustering. MLMF initially processes multi-omics feature matrices by performing multi-layer linear or nonlinear factorization, decomposing the original data into latent feature representations unique to each omics type. These latent representations are subsequently fused into a consensus form, on which spectral clustering is performed to determine subtypes. Additionally, MLMF incorporates a class indicator matrix to handle missing omics data, creating a unified framework that can manage both complete and incomplete multi-omics data. Extensive experiments conducted on 12 multi-omics cancer datasets, both complete and with missing values, demonstrate that MLMF achieves results that are comparable to or surpass the performance of several state-of-the-art approaches. MLMF is open source and available at (https://github.com/renyingxuan/MLMF.git).

## Introduction

Cancer is one of the main global health threats, with high rates of incidence and mortality that make it a focal point of current medical research and public health efforts. Its occurrence and development are a biological change with a complex mechanism. Different subtypes of the same cancer can differ in histopathology and clinical features, but the heterogeneity of cancer is mainly due to its intrinsic molecular characteristics [1]. Therefore, making full use of the intrinsic molecular characteristics of cancer to identify cancer subtypes will help to achieve precision medicine for cancer. In precision medicine, the molecular profile of a patient contains multiple molecules that belong to different omics (such as genomics, proteomics, metabolomics, etc.). These omics data reflect different biological processes, such as gene expression, protein function, metabolic pathways, etc. Early studies usually conducted statistics and research on a single omics datasets [2]. However, single omics data can only reflect the cancer characteristics of a certain level of biological process [3], and using different single omics data to address the same question can produce different results. For a heterogeneous disease such as cancer, its occurrence and development are affected by different gene combinations and various factors, so using single omics data cannot fully describe the complete information of cancer [4]. Different omics data are combined to describe the patient’s biological information, which is called “multi-omics data” [5]. Currently, common multi-omics data includes CNV, mRNA expression, miRNA expression, DNA methylation, etc. [6].

Nowadays, cancer subtype identification based on multi-omics data is mainly achieved through the integrated analysis of cancer sample data [7]. The current methods can be roughly divided into three categories: early integration, mid-term integration, and late integration [8]. For early integration, the main principle is to concatenate the input feature matrices of different omics into a multi-omics feature matrix, and then apply traditional clustering algorithms such as K-means, spectral clustering, etc. on the multi-omics feature matrix [9]. Through clustering, each category corresponds to a different cancer subtype. For example, LRAcluster [10] is an integrated probability model based on low-rank approximation. It finds the global optimal solution of the objective function through a simple gradient ascent algorithm, and then uses the K-means method on the latent representation matrix to obtain the results of cancer subtypes [11]. For early integration, data fusion is achieved by direct splicing, hence the integrating process cannot reflect on the correlation between different omics. However, due to overly simple operations, the spliced data often contains redundant information, which increases the data dimension of the input model. The main principle of late integration is to use the clustering algorithm of a single omics on each omics separately, and then integrate the different clustering results obtained from all omics as the final identification result [12]. The PINS method [13] constructs a connectivity matrix by integrating the clustering results of various omics data and integrates the connectivity matrix into a similarity matrix for clustering. The CC algorithm [14] verifies the rationality of clustering by randomly extracting subsets from the original data, specifying the number of clusters, and clustering all data subsets separately. Although the late integration method does not increase the data dimension of the input model, it can adopt a single omics normalization for each data type and use a model adapted to each omics data, but it cannot establish inter-omics associations at the feature level. Mid-term integration is the most common mainstream method, which globally involves data integration in a learning process. The MCCA algorithm [15] uses sparse canonical correlation analysis to find highly correlated omics data. iClusterBayes [16] based on iCluster uses a full Bayesian latent variable model to select valuable latent variables and describe the intrinsic structure in multi-omics data. Xu *et al.* [17] proposed the MSNE algorithm to integrate multi-omics information by embedding similarity relationships of samples defined by random walks on multiple similarity networks.

Another problem with using multi-omics data to identify cancer subtypes is that the high cost of sequencing technology can lead to incomplete multi-omics data. Some patients may only have their mRNA expression data or DNA methylation data sequenced. In this case, there is no complete available multi-omics data. If a complete clustering algorithm based on multi-omics data is used in incomplete samples, it will inevitably fail and affect the performance of clustering. Some methods proposed recently have begun to address the problem of incomplete data. NEMO [18] allows samples to be missing in one or more datasets. If each pair of samples has a measurement value in at least one omics dataset, cancer subtypes can be identified. MSNE [17] captures the comprehensive similarity of samples by random walks on multiple similarity networks and is also applicable when data is missing. Therefore, how to effectively use these incomplete multi-omics data to better identify cancer subtypes has become an important issue in this research field.

The semi-non-negative matrix factorization is a commonly used representation learning method, and currently, some studies have attempted to use this method to solve bioinformatics problems, such as pathways identification [19], drug–drug interactions prediction [20], gene representation analysis [21], etc. Therefore, a Multi-Layer Matrix Factorization method called MLMF for cancer subtyping via multi-omics data clustering is proposed in this paper. MLMF first takes the feature matrix of multi-omics as input, performs multi-layer linear or nonlinear factorization on the matrix, decomposes the original multi-omics data representation into their respective latent feature representations, and then fuses these representations into a consensus representation. Finally, spectral clustering is performed on this consensus representation. In addition, an indicator matrix is used to represent the missing status of some samples in the omics, thereby unifying the processes of complete multi-omics and missing multi-omics in a common framework.

## Method

MLMF obtains consensus representation and then cancer subtyping is carried out on the consensus representation via spectral clustering algorithm [22].

### Notation

Let $\boldsymbol{X}=\{\boldsymbol{X}^{(1)},\boldsymbol{X}^{(2)},\ldots ,\boldsymbol{X}^{(V)}\}$ represents multi-omics dataset, where $V$ is the number of omics. $\boldsymbol{X}^{(v)}=\left \{\boldsymbol{x}_{1}^{(v)},\boldsymbol{x}_{2}^{(v)},...,\boldsymbol{x}_{N_{v}}^{(v)}\right \}\in \mathbb{R}^{D_{v}\times N_{v}}$ is a collection of $N_{v}$ data samples with dimension $D_{v}$ in $vth$ omics measurements, where $v=1,2,\ldots ,V$. The consensus representation is $\boldsymbol{H}=\left \{\boldsymbol{h}_{1},\boldsymbol{h}_{2},\ldots ,\boldsymbol{h}_{N}\right \}^{T}\in \mathbb{R}^{N\times d}$, where $d$ is the ultimate dimension of consensus embedding space and $N\ (N\geq N_{v})$ is the sample size of total data. ${\parallel \cdot \parallel }_{F}^{2}$ is the Frobenius norm.

Since data may be missing, the sample index matrix $\boldsymbol{G}^{\left (v\right )}$ on each omics data is constructed as follows:


(1) \begin{align*}& \boldsymbol{G}_{ij}^{(v)} = \begin{cases} 1, & \text{if}\ \ i\text{th sample in} \ \boldsymbol{X}^{(v)} \ \text{ is the} \ j \text{th sample in intact data} \\ 0, & \text{otherwise} \end{cases}\end{align*}

### The framework of MLMF

**Figure 1:**
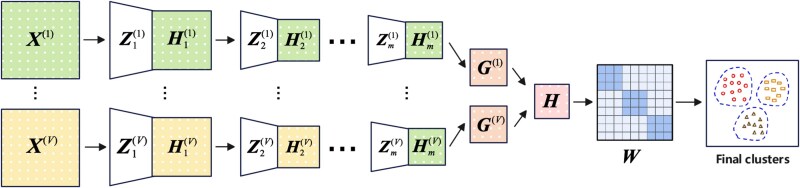
The framework of MLMF.

As shown in Fig. 1, MLMF mainly contains two modules. First, the deep semi-non-negative matrix factorization algorithm is used to perform multi-layer factorization of each omics data to obtain a deep low-dimensional representation. According to the mapping way, it can be formulated two strategies: linear mapping and nonlinear mapping. Then in the consensus representation module, indicator matrix is used to represent the missing status of some samples in the omics, and then fuses these representations into a consensus representation. The consensus representation retains as much original information as possible through the minimum reconstruction loss. Finally, cancer subtype is identified on consensus representation via spectral clustering. MLMF employs a matrix factorization approach to derive intermediate representations for each omics data type, subsequently solving an optimization problem to integrate these representations into a unified form. Therefore, MLMF belongs to mid-term integration methods in multi-omics data analysis.

### Linear MLMF

The optimization problem based on the deep semi-nonnegative matrix factorization can be written as follows.


(2) \begin{align*}& \begin{aligned} & \min_{\boldsymbol{Z}_{i}^{(v)}, \boldsymbol{H}_{m}^{(v)}} \sum_{v=1}^{V} \left(\left\| \boldsymbol{X}^{(v)} - \boldsymbol{Z}_{1}^{(v)} \boldsymbol{Z}_{2}^{(v)} \ldots \boldsymbol{Z}_{m}^{(v)} \boldsymbol{H}_{m}^{(v)} \right\|_{F}^{2} \right. \\ & \left. +\ \textstyle \sum_{j} \left\| (\boldsymbol{H}_{m}^{(v)})_{.j} \right\|_{1}^{2} \right) \\ & \text{s.t.}\ \boldsymbol{H}_{m}^{(v)} \geq 0 \end{aligned}\end{align*}

Among them, $\boldsymbol{H}_{m}^{\left (v\right )}$ is the $\mathit{m}$ layer embedding representation of the $\mathit{v}$th omocs data, and $\boldsymbol{Z}_{i}^{(v)}$ is the $i$th layer basis matrix. The $\sum _{j}\left \|(\boldsymbol{H}_{m}^{(v)})_{.\mathrm{\mathit{j}}}\right \|_{1}^{2} $ module is used to control the sparsity of $\boldsymbol{H}_{m}^{\left (v\right )}$, and the specific formula is as follows:


(3) \begin{align*}& \textstyle\sum_{j}\left\|\left(\boldsymbol{H}_{m}^{(v)}\right)_{\cdot j}\right\|_{1}^{2}=Tr\left[\left(\boldsymbol{H}_{m}^{(v)}\right)\left(\boldsymbol{H}_{m}^{(v)}\right)^{T}\boldsymbol{E}\right]\end{align*}

$\boldsymbol{E}$ is a matrix with all elements equal to 1, and $Tr(\cdot )$ represents the trace operation of the matrix. The final consistent representation should consider the missing status of some samples, thus it:


(4) \begin{align*}& {\hat{\boldsymbol{H}}}_{m}^{\left(v\right)}=\boldsymbol{H}\boldsymbol{G}^{\left(v\right)}\end{align*}

Among them, $\boldsymbol{G}^{(v)}$ is the index matrix that records the missing data. By minimizing the reconstruction error, the purpose of optimizing the consensus representation $\boldsymbol{H}$ and the deep feature matrix $\boldsymbol{H}_{m}^{\left (v\right )}$ of each omics data can be achieved. So the optimization goal of the reconstruction stage is defined as follows:


(5) \begin{align*}& \min_{\boldsymbol{H}_{m}^{(v)},\boldsymbol{H}} \sum_{v=1}^{V} \left\| \boldsymbol{H}_{m}^{(v)} - \boldsymbol{H}\boldsymbol{G}^{(v)} \right\|_{F}^{2}.\end{align*}

To sum up, the overall optimization object of linear MLMF can be written as:


(6) \begin{align*}& \begin{aligned} & \min_{\boldsymbol{Z}_{i}^{(v)},\boldsymbol{H}_{m}^{(v)},\boldsymbol{H}} \sum_{v=1}^{V} \left( \left\|\boldsymbol{X}^{(v)} - \boldsymbol{Z}_{1}^{(v)}\boldsymbol{Z}_{2}^{(v)} \dots \boldsymbol{Z}_{m}^{(v)} \boldsymbol{H}_{m}^{(v)} \right\|_{F}^{2} \right. \\ & \left. +\ \lambda_{1} \textstyle \sum_{j} \left\| \left( \boldsymbol{H}_{m}^{(v)} \right)_{.j} \right\|_{1}^{2} + \lambda_{2} \left\| \boldsymbol{H}_{m}^{(v)} - \boldsymbol{HG}^{(v)} \right\|_{F}^{2} \right) \\ & \text{s.t.}\ \boldsymbol{H}_{m}^{(v)} \geq 0. \end{aligned}\end{align*}

Among them, $\lambda _{1}$ and $\lambda _{2}$ are penalty trade-off coefficients.

The problem is solved using the coordinate-descent iterative algorithm. The detailed solution process for each variable is shown in Supplementary Note 1.

For $\boldsymbol{Z}_{i}^{(v)}\ (1\le i\le m)$, it is updated as follows:


(7) \begin{align*}& \boldsymbol{Z}_{i}^{\left(v\right)}=\boldsymbol{X}\boldsymbol{\phi}^{-1}\boldsymbol{X}^{\left(v\right)}(\boldsymbol{H}_{i}^{\left(v\right)})^{-1}\end{align*}

where $\boldsymbol{\phi }=\boldsymbol{Z}_{1}^{\left (v\right )}\boldsymbol{Z}_{2}^{\left (v\right )}...\boldsymbol{Z}_{i-1}^{(v)}$ and $\boldsymbol{H}_{i}^{(v)}=\boldsymbol{Z}_{i+1}^{(v)}...\boldsymbol{Z}_{m}^{(v)}\boldsymbol{H}_{m}^{(v)}$.

For $\boldsymbol{H}_{m}^{(v)}$, it is updated as follows:


(8) \begin{align*}& \boldsymbol{A}_{ik}\gets\boldsymbol{A}_{ik}\sqrt{\frac{\boldsymbol{B}_{ik}^{+}+(\boldsymbol{C}^{-}\boldsymbol{A})_{ik}}{\boldsymbol{B}_{ik}^{-}+(\boldsymbol{C}^{+}\boldsymbol{A})_{ik}}}.\end{align*}

where $\boldsymbol{A}=\left (\boldsymbol{H}_{m}^{\left (v\right )}\right )$, and $\boldsymbol{I}$ is the unit matrix. $\boldsymbol{B}=\boldsymbol{\Psi }_{m}^{T}\boldsymbol{X}^{\left (v\right )}+\lambda _{2}\boldsymbol{H}\boldsymbol{G}^{\left (v\right )}$, $\boldsymbol{C}=\boldsymbol{\Psi }_{m}^{T}\boldsymbol{\Psi }_{m}+\lambda _{1}\boldsymbol{E}+\lambda _{2}\boldsymbol{I}$.

For $\boldsymbol{H}_{i}^{\left (v\right )}\ (i<m)$, it is updated as follows:


(9) \begin{align*}& \boldsymbol{H}_{ik}^{\left(v\right)} \gets \boldsymbol{H}_{ik}^{\left(v\right)} \sqrt{\frac{(\boldsymbol{\Psi}_{i}^{T} \boldsymbol{X}^{\left(v\right)})_{ik}^{+} + \left(\left(\boldsymbol{\Psi}_{i}^{T} \boldsymbol{\Psi}_{i}^{-}\right)\boldsymbol{H}\right)_{ik}} {(\boldsymbol{\Psi}_{i}^{T} \boldsymbol{X}^{\left(v\right)})_{ik}^{-} + \left(\left(\boldsymbol{\Psi}_{i}^{T} \boldsymbol{\Psi}_{i}^{-}\right)\boldsymbol{H}\right)_{ik}}}\end{align*}

where $\boldsymbol{\Psi }_{i}=\boldsymbol{Z}_{1}^{\left (v\right )}\boldsymbol{Z}_{2}^{\left (v\right )}\ldots \boldsymbol{Z}_{i}^{\left (v\right )}$.

For $\boldsymbol{H}$, it is updated as follows:


(10) \begin{align*}& \boldsymbol{H} = \sum_{v=1}^{V} \; \boldsymbol{H}_{m}^{(v)} \boldsymbol{G}^{(v)T} \left( \sum_{v=1}^{V} \; \boldsymbol{G}^{(v)} \boldsymbol{G}^{(v)T} \right)^{-1}.\end{align*}

Summarizing the above steps, the optimization process of the Linear MLMF is shown in Algorithm 1.



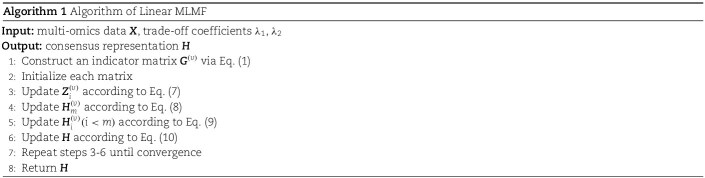



### Nonlinear MLMF

By linearly decomposing the initial data distribution, it may not be possible to effectively describe the nonlinear relationship between the omics data. Hence, we introduce the Nonlinear MLMF.

First, construct the loss function. Compared with linear factorization, nonlinear factorization uses nonlinear mapping in all factorizations except the first layer. Nonlinear factorization decomposes the given data matrix $\boldsymbol{X}$ into $m+1$ factors in a nonlinear way, as $\boldsymbol{X}\approx \boldsymbol{Z}_{1}f(\boldsymbol{Z}_{2}f(...f(\boldsymbol{Z}_{m}\boldsymbol{H}_{m}^{+})))$. $\boldsymbol{H}_{m}^{+}$ is the m-level implicit representation of the data, which can be given by the following factorization:


(11) \begin{align*}&\begin{aligned} \boldsymbol{H}_{m-1}^{+}& \approx f(\boldsymbol{Z}_{m}\boldsymbol{H}_{m}^{+}). \\ \end{aligned}\end{align*}

The optimization goal of the deep matrix nonlinear factorization model is as follows:


(12) \begin{align*}& \begin{aligned} \min_{\boldsymbol{Z}_{i}^{(v)},\boldsymbol{H}_{m}^{(v)},\boldsymbol{H}} L = & \sum_{v=1}^{V} \left\| \boldsymbol{X}^{(v)} - \boldsymbol{Z}_{1}^{(v)} f \left( \boldsymbol{Z}_{2}^{(v)} f \left( \ldots f \left( \boldsymbol{Z}_{m}^{(v)} \boldsymbol{H}_{m}^{(v)} \right) \right) \right) \right\|_{F}^{2} \\ & \quad + \lambda_{1} \textstyle \sum_{j} \left\| \left( \boldsymbol{H}_{m}^{(v)} \right)_{.j} \right\|_{1}^{2} + \lambda_{2} \left\| \boldsymbol{H}_{m}^{(v)}-\boldsymbol{HG}^{(v)} \right\|_{F}^{2} \\ & \text{s.t.}\ \boldsymbol{H}_{m}^{(v)} \geq 0. \end{aligned}\end{align*}

The problem is solved using the gradient descent method. The detailed solution process for each variable is shown in the Supplementary Note 2.

For $\boldsymbol{H}_{i}^{(v)}\ (1\le i\le m)$, it is updated as follows:


(13) \begin{align*}& \boldsymbol{H}_{i}^{\left(v\right)}=\boldsymbol{H}_{i}^{\left(v\right)}-\alpha\frac{\partial L}{\partial\boldsymbol{H}_{i}^{\left(v\right)}}\end{align*}

where $\frac{\partial L}{\partial \boldsymbol{H}_{i}^{(v)}}=\boldsymbol{Z}_{i}^{(v)^{T}}\left [\frac{\partial L}{\partial \boldsymbol{H}_{i-1}^{(v)}}\odot \nabla f\left (\boldsymbol{Z}_{i}^{(v)}\boldsymbol{H}_{i}^{(v)}\right )\right ]$ and $\frac{\partial L}{\partial \boldsymbol{H}_{1}^{(v)}}=\boldsymbol{Z}_{1}^{(v)^{T}}\!\Big(2\boldsymbol{Z}_{1}^{(v)}\boldsymbol{H}_{1}^{(v)} {-} 2\boldsymbol{X}^{(v)}\Big)$

For $\boldsymbol{Z}_{i}^{(v)}\ (1\le i\le m)$, it is updated as follows:


(14) \begin{align*}& \boldsymbol{Z}_{i}^{\left(v\right)}=\boldsymbol{Z}_{i}^{\left(v\right)}-\alpha\frac{\partial L}{\partial\boldsymbol{Z}_{i}^{\left(v\right)}}\end{align*}

where $\frac{\partial L}{\partial \boldsymbol{Z}_{i}^{(v)}}=\left [\frac{\partial L}{\partial \boldsymbol{H}_{i-1}^{(v)}}\odot \nabla f\left (\boldsymbol{Z}_{i}^{(v)}\boldsymbol{H}_{i}^{(v)}\right )\right ]\boldsymbol{H}_{i}^{(v)^{T}}$ and $\frac{\partial L}{\partial \boldsymbol{Z}_{1}^{(v)}}=2\Big(\boldsymbol{Z}_{1}^{(v)}\boldsymbol{H}_{1}^{(v)}-\boldsymbol{X}^{(v)}\Big)\boldsymbol{H}_{1}^{(v)^{T}}$

For $\boldsymbol{H}$, it is updated as follows:


(15) \begin{align*}& \boldsymbol{H}=\boldsymbol{H}-\alpha\frac{\partial L}{\partial\boldsymbol{H}}\end{align*}

where $\frac{\partial L}{\partial \boldsymbol{H}}=\partial \lambda _{2}\sum _{v=1}^{V}\left (-2\boldsymbol{H}_{m}^{(v)^{T}}\boldsymbol{G}^{(v)}+\boldsymbol{HG}^{(v)}\boldsymbol{G}^{(v)^{T}}\right )$

The optimization process of the deep matrix nonlinear factorization algorithm is shown in Algorithm 2.



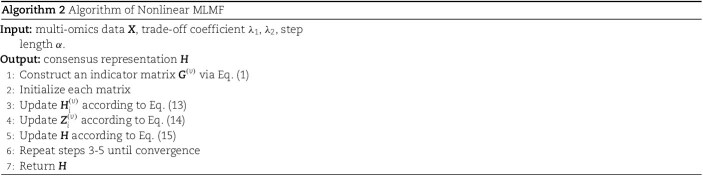



Finally, the similarity matrix $\boldsymbol{W}$ is constructed as follows:


(16) \begin{align*}& \begin{aligned} \boldsymbol{W}_{ij}= \begin{cases} \exp\left(-\left\|\boldsymbol{h}_{i}-\boldsymbol{h}_{j}\right\|_{2}^{2}/t\right),{\rm if}\ \boldsymbol{h}_{i}\in Nei(\boldsymbol{h}_{j})\ or\ \boldsymbol{h}_{j}\in Nei(\boldsymbol{h}_{i}) \\ \\ 0,{\rm otherwise} & \end{cases} \end{aligned}\end{align*}

where $t$ is a tuning parameter and $Nei$ is the set of neighborhoods.

## Results

### Full muti-omics datasets

Several computational experiments evaluate the effectiveness of cancer subtypes with multi-omics data. This paper conducted experiments on 11 cancer datasets (AML, BIC, COAD, GBM, KIRC, LIHC, LUSC, OV, SKCM, SARC, and HNSC) from TCGA [23] and the METABRIC dataset. TCGA datasets include mRNA expression, DNA methylation and miRNA expression data. METABRIC dataset only contains mRNA expression and CNV data. The feature data after dimensionality reduction is standardized using z-score. All data is preprocessed is the same as in Rappoport and Shamir [18]. The detailed information about the processed datasets is shown in Supplementary Table 1.

This article compares MLMF with 11 selected algorithms on complete multi-omics datasets, including K-means and spectral clustering algorithms, as well as 9 integration methods such as LRAcluster [10], PINS ([13], MCCA [15], iClusterBayes [24], SNF [25], CC [14], SNFCC [26], NEMO [18], and IntNMF [27]. The evaluation indicators used for the identified subtype results are the enrichment number of clinical parameters and the significance of survival analysis. The number of subtypes for each cancer type was determined by feature factorization. For simplicity, the penalty coefficients $\lambda _{1}$ and $\lambda _{2}$ are both set to 1, and the step size is adjusted in an adaptive manner. In the construction of the similarity matrix $\boldsymbol{W}$, the adjustment parameter $t> 0$ is used to control the scale of the similarity calculation. The maximum number of iterations is set to 50, and the convergence tolerance $TolFun$ is $1e-4$ to balance the computational efficiency and result accuracy. Survival analysis using the Cox proportional hazards model and p-value showed statistically significant differences in the survival spectra of different cancer subtypes [28]. To perform enrichment analysis of clinical signatures, we selected a unified set of patient clinical information for all cancers, such as sex and age at initial diagnosis, as well as quantifying tumor progression (pathology T), lymph node cancer (pathology N), metastasis (pathology M) and overall progression (pathological stage) as four discrete clinicopathological parameters. Following the recommendations of Rappoport and Shamir (2019), the number of clusters in the comparison method was set to the same value as reported in the original paper.

**Figure 2:**
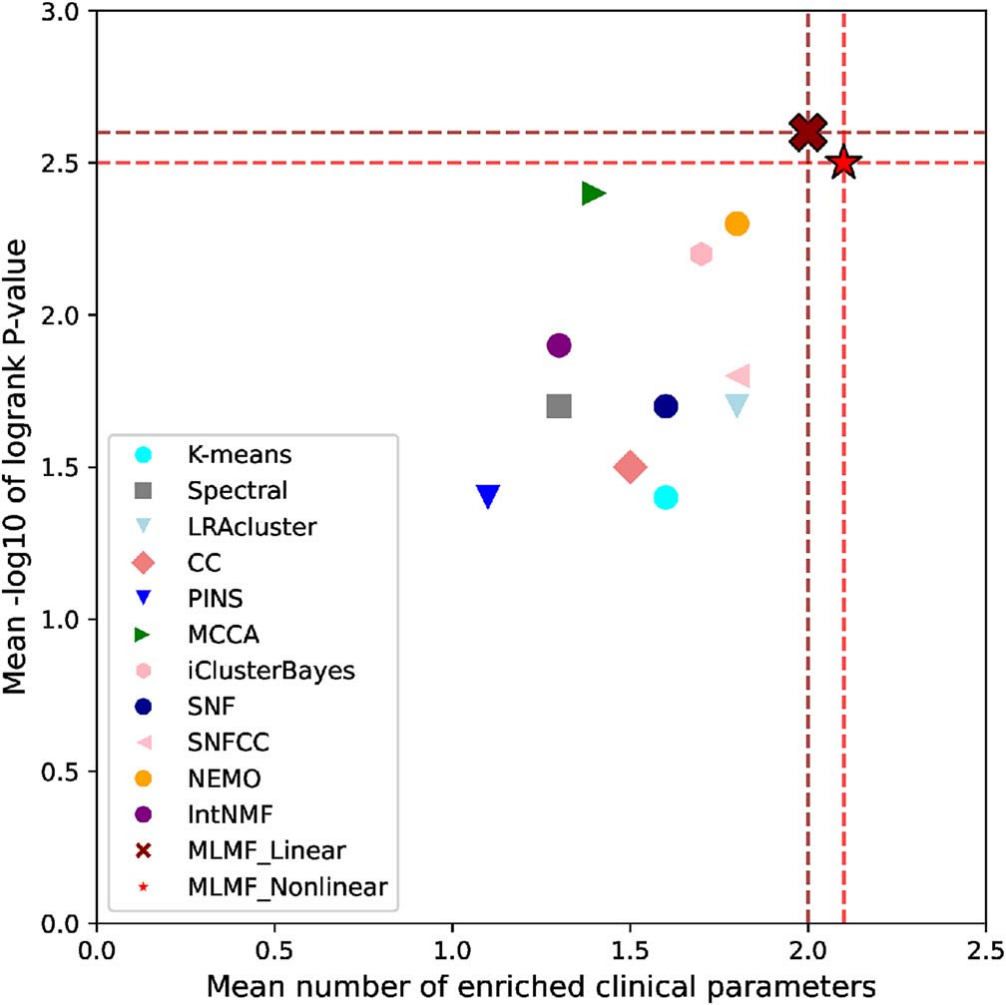
Mean performance of the different algorithms on 12 cancer datasets.

Table 1 and Fig. 2 show the cancer subtype prediction performance of different algorithms on 12 complete TCGA datasets. As can be seen from the results, the clusters discovered by MLMF_Linear and MLMF_nonLinear had significant survival differences in 10 of the 12 cancer datasets. The average logrank p-value of MLMF_Nonlinear reaches 2.5, and the average logrank p-value of MLMF_Linear reaches 2.6. MCCA ranked third with 2.4. None of the methods found significant differences in survival rates for the COAD dataset. MLMF_Linear and MLMF_Nonlinear found at least one enriched clinical parameter in all datasets. The average number of enriched clinical parameters for MLMF_Nonlinear was 2.1, and the average number of enriched clinical parameters for MLMF_Linear was 2.0. These results show that linear factorization and nonlinear factorization of MLMF can identify patient subtypes with significant consistency and clinical relevance.

**Table 1:** The comparison of clustering results from different algorithms on 12 full datasets

Alg./Cancer	AML	BIC	COAD	GBM	KIBC	LIHC	LUSC	OV	SKCM	SARC	HNSC	METABRIC	Mean	Sig
K-means	**1**/**2.4**	**2**/**3.5**	**1**/0.4	**2**/**2.6**	**1**/0.8	**2**/0.2	0/**1.5**	**2**/0.3	**2**/0.9	**2**/**1.3**	**2**/**1.7**	**2**/**1.4**	**1.6**/**1.4**	11/7
Spectral	**1**/**2.1**	**1**/**5.0**	**1**/0.7	**2**/**2.5**	**2**/**1.8**	**2**/0.4	0/**2.1**	**2**/0.8	0/0.6	**2**/**1.3**	**1**/**1.5**	**2**/**1.8**	**1.3**/**1.7**	10/8
LRAcluster	**1**/**1.8**	**2**/**4.0**	**1**/0.1	**2**/1.1	**2**/1.0	**2**/**2.4**	**1**/1.0	**2**/0.2	**3**/**2.9**	**2**/**2.5**	**2**/**1.6**	**2**/**2.0**	**1.8**/**1.7**	12/7
CC	**1**/**3.8**	**1**/**2.8**	**1**/0.5	**2**/**2.1**	**3**/**1.3**	**2**/0.5	**1**/1.1	**1**/0.2	**3**/**2.5**	**2**/1.0	0/1.1	**1**/**1.3**	**1.5**/**1.5**	11/6
PINS	**1**/**1.6**	**1**/**2.8**	0/0.5	**1**/**4.4**	**2**/1.0	**2**/0.8	0/**1.9**	**1**/0.1	**1**/1.0	**2**/0.8	**1**/0.9	**1**/1.2	**1.1**/**1.4**	10/4
MCCA	**1**/1.2	**1**/**8.0**	0/0.2	**1**/**2.9**	**2**/**1.8**	**2**/1.1	**2**/**2.3**	0/0.6	**2**/**4.7**	**2**/**1.5**	**2**/**2.1**	**2**/**2.4**	**1.4**/**2.4**	10/8
iClusterBayes	**1**/**1.5**	0/**1.3**	**2**/0.1	**1**/**3.1**	**4**/**7.3**	**2**/**2.2**	0/**1.5**	**2**/0.9	**2**/0.6	**2**/**3.7**	**2**/**1.5**	**2**/**2.3**	**1.7**/**2.2**	10/9
SNF	**1**/**3.0**	**2**/**6.0**	**1**/0.2	**2**/**2.6**	**3**/**1.7**	**2**/0.3	**1**/1.2	**2**/0.2	**1**/1.1	**2**/**1.9**	**1**/1.2	**1**/**1.3**	**1.6**/**1.7**	12/6
SNFCC	**1**/**3.8**	**3**/**7.2**	**2**/0.6	**2**/**2.3**	**2**/1.1	**1**/1.2	**1**/1.0	**1**/0.2	**2**/0.6	**2**/1.1	**2**/**1.4**	**2**/**1.6**	**1.8**/**1.8**	12/5
NEMO	**1**/**1.8**	**2**/**4.2**	0/0.1	**1**/**3.8**	**4**/**2.2**	**4**/**4.2**	0/**1.8**	**1**/0.4	**3**/**4.0**	**2**/**1.9**	**2**/**1.3**	**2**/**1.5**	**1.8**/**2.3**	10/10
IntNMF	**1**/**1.9**	**1**/**4.3**	**1**/0.2	**1**/**3.5**	**3**/0.2	**2**/**2.0**	0/0.9	0/0.7	**2**/**4.1**	**2**/**1.8**	**2**/**1.7**	**1**/**1.9**	**1.3**/**1.9**	10/8
MLMF_Linear	**1**/**3.4**	**3**/**5.9**	**1**/0.4	**2**/**4.1**	**2**/**1.4**	**2**/**3.2**	**1**/**1.8**	**2**/**1.9**	**3**/**2.9**	**2**/1.0	**2**/**2.2**	**3**/**2.6**	**2.0**/**2.6**	12/10
MLMF_Noninear	**1**/**3.1**	**4**/**5.5**	**2**/0.3	**1**/**4.5**	**3**/**1.5**	**3**/**3.1**	**1**/**1.6**	**1**/**2.7**	**3**/**4.3**	**2**/0.8	**2**/**1.3**	**2**/**1.5**	**2.1**/**2.5**	12/10

Note: in each cell A/B, A is significant clinical parameters detected. B is -log10 P-value for survival. 0.05 is the threshold for significance and the bold indicates the significant results. Mean is algorithm average value. Sig is the number of datasets with significant results.

**Figure 3:**
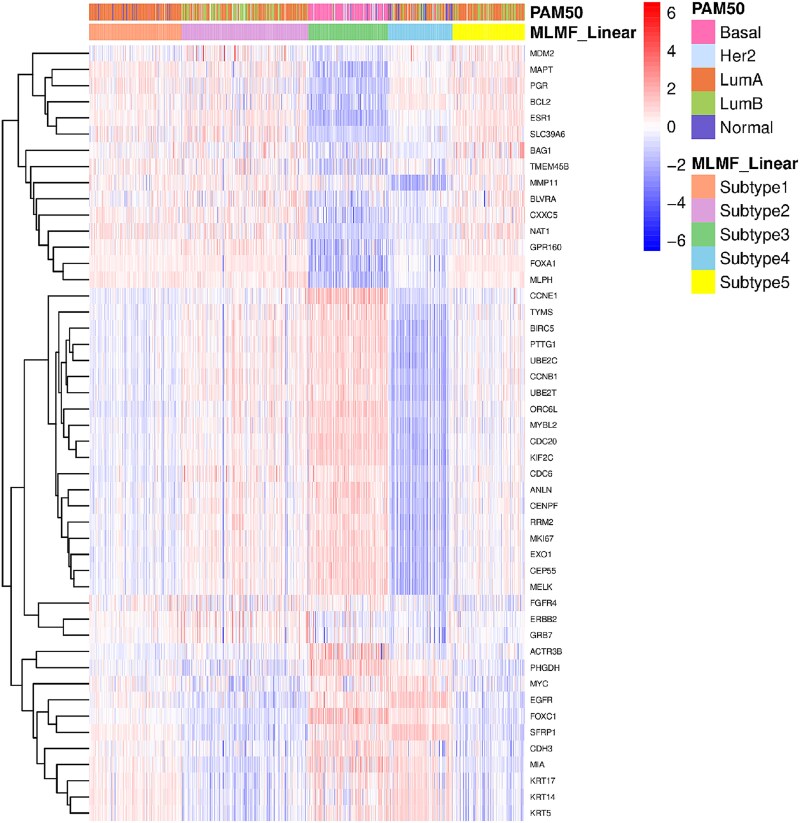
The heatmap for BIC dataset.

In order to verify the subtypes obtained by MLMF_Linear and the existing subtypes, and to show the differential expression between different subtypes, this paper designed the following experiments. First, the subtype results of PAM50 on the BIC dataset were selected for comparison. Secondly, since there were 48 mRNA expression features associated with the 50 genes of PAM50, we deleted the 48 features in the original mRNA data of the BIC dataset to eliminate the direct effects of known oncogenes in multi-omics data, and then input the processed mRNA data into MLMF_Linear together with other omics data. Finally, a heat map was drawn using the expression of the 48 mRNAs to show the correlation between oncogenes and subtypes obtained from MLMF_Linear, as well as the overlap of subtypes obtained by MLMF_Linear and PAM50. As shown in Fig. 3, different subtypes have different mRNA expression patterns, and there is a large overlap between MLMF_Linear and PAM50, such as the LumA subtype of PAM and subtype 1 of MLMF_Linear, and the Basal subtype of PAM and subtype 3 of MLMF_Linear.

**Figure 4:**
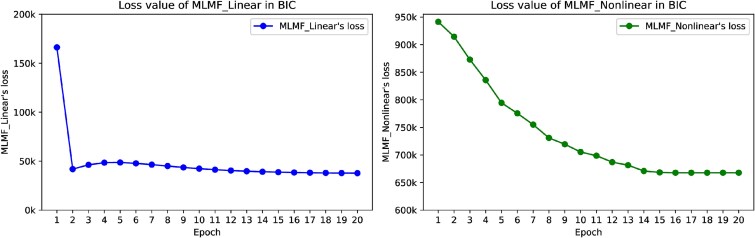
The change of the loss function values of MLMF_Linear and MLMF_Nonlinear under 20 epochs.

In order to verify the training effect of the MLMF algorithm, this paper records the changes in the loss function values of MLMF_Linear and MLMF_Nonlinear under 20 epochs, as shown in Fig. 4. It can be seen from the figure that the loss of MLMF_Linear and MLMF_Nonlinear both show a downward and convergent trend. MLMF_Linear has a great improvement in the early stage of training, and the loss drops rapidly. The convergence process of MLMF_Nonlinear is more stable, showing a gradual downward trend. Since the analytical solution at each iteration in coordinate descent of the linear MLMF could be obtained, the linear method had a faster convergence rate [29].

To verify that the cancer subtypes identified by the MLMF_Linear algorithm are biologically meaningful and provide interpretability, we performed GO enrichment analysis on the experimental data results, as detailed in Supplementary Note 3. The analysis results showed that different cancer subtypes exhibited significant differences in key biological processes.

### Partial multi-omics datasets

To evaluate the performance of the method on some multi-omics datasets, this paper still selected the 12 datasets analyzed above and simulated some patient loss omics measurements. Specifically, TCGA datasets maintain the complete expression of DNA methylation and miRNA, and randomly extracts samples from a part of patients to remove their mRNA expression, with missing rates of 0.1, 0.3, 0.5, and 0.7. For METABRIC dataset, maintain CNV and remove mRNA. Enrichment analysis and survival analysis are still used to evaluate the performance of the method. Table 2 shows the comparison results of different algorithms on 12 simulated missing datasets.

**Table 2:** Performance of different algorithms on 12 simulated missing datasets

Alg./Cancer	AML	BIC	COAD	GBM	KIBC	LIHC	LUSC	OV	SKCM	SARC	HNSC	METABRIC	Mean	Sig
						$\theta $ = 0.1								
MCCA	**1**/**3.9**	**1**/**3.5**	0/0.2	**1**/**2.0**	**2**/**2.2**	**1**/0.7	0/0.9	**2**/0.3	**1**/**2.7**	0/0.8	**1**/**1.5**	**1**/**1.7**	**0.9**/**1.7**	9/7
NEMO	**1**/**3.1**	**2**/**4.3**	**1**/0.1	**1**/**2.8**	**3**/**1.5**	**3**/**2.8**	**1**/**2.2**	**1**/0.1	**1**/0.5	**2**/0.9	**2**/1.1	**2**/**1.6**	**1.7**/**1.8**	12/7
MLMF_Linear	**1**/**3.4**	**2**/**5.0**	**1**/0.7	**2**/**3.3**	**4**/**2.4**	**3**/**1.4**	**1**/**1.3**	**1**/**1.4**	**1**/**3.7**	**2**/0.8	**2/1.5**	**2/1.9**	**1.8**/**2.2**	12/10
MLMF_Noninear	**1**/**3.0**	**2**/**5.8**	**1**/**1.3**	**2**/**2.9**	**4**/**2.4**	**3**/**1.8**	**1**/**2.6**	**1**/**1.9**	**2**/**1.4**	**2**/0.6	**2/2.3**	**2/2.5**	**1.9**/**2.4**	12/11
						$\theta $ = 0.3								
MCCA	**1**/**2.0**	**2**/**3.6**	0/0.2	0/0.4	0/**1.5**	**1**/1.1	0/0.5	**2**/0.1	0/**1.7**	0/0.9	0/0.8	0/1.2	0.5/1.2	4/4
NEMO	**1**/**2.4**	**2**/**4.0**	**1**/0.3	**1/1.6**	**3**/1.2	**3**/**3.8**	0/0.7	**2**/0.3	0/0.2	**2**/0.9	**2/1.3**	**2/1.7**	**1.6**/**1.5**	10/6
MLMF_Linear	**1**/**2.4**	**2**/**5.3**	**1**/0.4	**2**/**3.7**	**2**/**1.9**	**1**/**2.0**	**1**/0.9	**2**/**1.4**	**1**/**1.4**	**2**/1.1	**2/1.4**	**2/1.7**	**1.6**/**2.0**	12/9
MLMF_Noninear	**1**/**2.5**	**2**/**5.6**	**1**/0.6	**2**/**3.5**	**3**/**1.9**	**1**/**2.2**	**1**/1.2	**2**/**2.7**	**2**/**2.2**	**2**/0.4	**2/2.5**	**2/2.3**	**1.8**/**2.3**	12/9
						$\theta $ = 0.5								
MCCA	**1**/**2.8**	**1**/**3.6**	0/0.3	**1**/**1.6**	**2**/**2.7**	**1**/0.6	0/0.8	**1**/0.1	**2**/1.1	**1**/**1.3**	**1**/1.0	**1/1.4**	**1.0**/**1.4**	10/6
NEMO	**1**/**3.1**	**2**/**4.7**	**1**/0.1	**1**/**2.7**	**1**/1.2	**2**/**1.9**	**1**/1.1	**1**/0.1	0/0.3	**2**/**2.1**	**1**/1.2	**2/2.1**	**1.3**/**1.7**	11/6
MLMF_Linear	**1**/**3.1**	**2**/**4.8**	**1**/0.3	**2**/**3.4**	**2**/**2.0**	**2**/**1.5**	**1**/**1.3**	**1**/0.9	0/0.5	**1**/1.0	**2/1.6**	**2/2.3**	**1.4**/**1.9**	11/8
MLMF_Noninear	**1**/**2.8**	**1**/**4.7**	**2**/0.4	**2**/**3.9**	**3**/**2.5**	**2**/**1.7**	**1**/0.8	**1**/0.7	0/**1.4**	**2**/0.7	**1/1.8**	**2/2.2**	**1.5**/**2.0**	11/8
						$\theta $ = 0.7								
MCCA	**1**/**2.1**	**1**/**3.8**	0/0.3	**1**/**2.5**	**2**/**2.6**	**1**/**1.3**	0/**1.3**	**1**/0.1	**2**/**2.4**	0/0.1	0/0.8	**1/2.1**	**0.8**/**1.6**	8/8
NEMO	**1**/**2.9**	**2**/**4.5**	**1**/0.1	**1**/**3.3**	**4**/**2.2**	**2**/**1.9**	0/1.1	**1**/0.1	0/0.3	**2**/0.9	**1**/1.1	**2/1.9**	**1.4**/**1.7**	10/6
MLMF_Linear	**1**/**2.6**	**2**/**4.8**	**1**/0.3	**2**/**3.6**	**3**/**1.7**	**1**/**1.7**	**2**/0.3	**1**/0.9	0/**1.4**	**1**/**1.9**	**1/1.6**	**2/1.8**	**1.4**/**1.9**	11/9
MLMF_Noninear	**1**/**2.9**	**2**/**4.4**	**1**/0.3	**2**/**3.1**	**3**/**2.7**	**2**/**2.1**	**1**/1.1	**2**/0.5	0/**1.4**	**1**/**1.9**	**1/1.8**	**2/2.4**	**1.5**/**2.1**	11/9

Note: $\theta $ is the fraction of missing data. In each cell A/B, A is significant clinical parameters detected. B is -log10 P-value for survival. 0.05 is the threshold for significance and the bold indicates the significant results. Mean is algorithm average value. Sig is the number of datasets with significant results.

**Figure 5:**
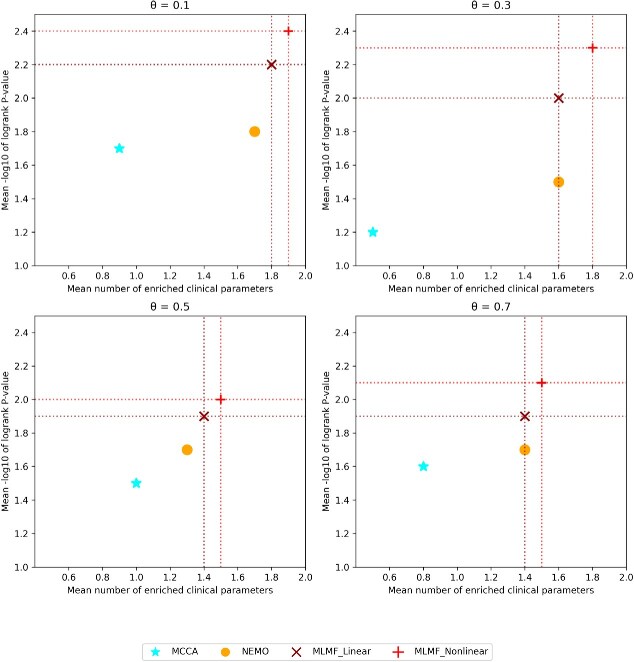
Mean performance of the different algorithms on 12 cancer datasets. $\theta $ is the fraction of missing data.

Table 2 and Fig. 5 show the cancer subtype prediction performance of different algorithms on 12 incomplete TCGA datasets. MLMF_Linear and MLMF_Nonlinear performed better than NEMO and MCCA in survival and enrichment analysis at all missing rates. Under the same missing rate, the average performance of the nonlinear decomposition algorithm is better than that of the linear decomposition. These results indicate that MLMF can be well applied to situations where part of the omics is missing. In general, cancer subtyping by MLMF resulted in statistically significant survival spectrum differences and significant clinical enrichment. In addition, MLMF can effectively solve the challenge of missing parts of the omics.

In order to evaluate the efficiency of the MLMF algorithm, we compared the running time of the MLMF_Linear algorithm and the MLMF_Nonlinear algorithm on the BIC dataset with ten algorithms, namely K-means, spectral clustering algorithms, LRAcluster, PINS, MCCA, iClusterBayes, SNF, SNFCC, and NEMO. As can be seen from Supplementary Fig. 2, the fastest algorithm is spectral clustering and the slowest algorithm is iClusterBayes. MLMF_Linear is at a medium level compared to the benchmark methods, and the running time of the MLMF_Nonlinear is still faster than CC and iClusterBayes. However, the cancer subtyping performance of MLMF is better than many state-of-the-art approaches.

## Conclusion

Predicting cancer subtypes using multi-omics data enables researchers and clinicians to adopt a more comprehensive and precise approach to patient treatment. Data from various omics offer distinct insights into biological processes, and by integrating these multi-omics datasets, researchers can uncover unique patterns and molecular features associated with different cancer subtypes. In this paper, we introduce MLMF, a multi-layer matrix decomposition method designed for cancer subtyping through the clustering of multi-omics data. For the first time, MLMF unifies the processing pipelines for complete and missing multi-omics data within a common framework. It performs multi-layer linear or nonlinear decomposition on the multi-omics feature matrix, breaking down the original data representation into respective latent feature representations. These representations are then fused to create a consensus representation. The identification of cancer subtypes is achieved through spectral clustering of this consensus representation. Experimental results from 12 multi-omics datasets demonstrate that MLMF outperforms other related methods. While our study focused on two to three omics levels, MLMF provides a versatile framework that can be easily adapted to scenarios involving additional omics data. We believe that MLMF holds significant promise for advancing precision oncology and enhancing patient outcomes.

**Key Points**

- A new multi-layer matrix factorization algorithm (MLMF) is proposed, which can simultaneously learn multi-omics feature representation and cancer subtype labels
- The identification process of complete and missing multi-omics data is unified in a common framework
- The experimental results on the TCGA datasets show that MLMF has advantages in the ability to identify cancer subtypes.

## Supplementary Material

Supplementary_Materials_for_MLMF_bbaf448
