# Supplementary material for: Multi-layer matrix factorization for cancer subtyping using full and partial multi-omics dataset
Source: Brief Bioinform. 2025 Sep 9;26(5):bbaf448. doi: 10.1093/bib/bbaf448 (PMC12418959; doi:10.1093/bib/bbaf448)
Supplement: Supplementary_Materials_for_MLMF_bbaf448 [file supplementary_materials_for_mlmf_bbaf448.pdf]

Supplementary Materials for "Multi-layer matrix factorization for cancer subtyping using full and partial multi-omics dataset"

Supplementary Note 1. Deep Matrix Linear Factorization

The overall optimization goal of the deep matrix linear factorization can be written as follows:

$$\min_{\boldsymbol{Z}_i^{(v)},\boldsymbol{H}_m^{(v)},\boldsymbol{H}} \sum_{v=1}^{V} \left(\left\| \boldsymbol{X}^{(v)} - \boldsymbol{Z}_1^{(v)}\boldsymbol{Z}_2^{(v)} \ldots \boldsymbol{Z}_m^{(v)}\boldsymbol{H}_m^{(v)} \right\|_F^2 + \lambda_1 \sum_j \left\| \left(\boldsymbol{H}_m^{(v)}\right)_{.j} \right\|_1^2 + \lambda_2 \left\| \boldsymbol{H}_m^{(v)} - \boldsymbol{H}\boldsymbol{G}^{(v)} \right\|_F^2 \right) \quad (1)$$
$$s.t. \boldsymbol{H}_m^{(v)} \geq 0$$

To solve the objective function, treat each variable as the only variable of the objective function, calculate its partial derivative and set it equal to 0. By updating the variables alternately, the optimal solution is solved.

For $\boldsymbol{Z}_i^{(v)}$ $(1 \leq i \leq m)$, fix the remaining variables and calculate $\boldsymbol{Z}_i^{(v)}$ by minimizing Eq. (2).

$$\mathcal{L} = \min_{\boldsymbol{Z}_i^{(v)}} \sum_{v=1}^{V} \left\| \boldsymbol{X}^{(v)} - \boldsymbol{Z}_1^{(v)}\boldsymbol{Z}_2^{(v)} \ldots \boldsymbol{Z}_m^{(v)}\boldsymbol{H}_m^{(v)} \right\|_F^2 \quad (2)$$

Let the derivative $\partial\mathcal{L}(\boldsymbol{Z}_i^{(v)})/\,\partial(\boldsymbol{Z}_i^{(v)}) = 0$. Since the $\boldsymbol{Z}_i^{(v)}$ of each omics data is different, the partial derivative can only be calculated on each omics data. The minimization of Eq. (2) is equivalent to the minimization Eq. (3).

$$\left\| \boldsymbol{X}^{(v)} - \boldsymbol{Z}_1^{(v)}\boldsymbol{Z}_2^{(v)} \ldots \boldsymbol{Z}_m^{(v)}\boldsymbol{H}_m^{(v)} \right\|_F^2 \quad (3)$$

Set $\phi = \boldsymbol{Z}_1^{(v)}\boldsymbol{Z}_2^{(v)} \ldots \boldsymbol{Z}_{i-1}^{(v)}$ and $\boldsymbol{H}_i^{(v)} = \boldsymbol{Z}_{i+1}^{(v)} \ldots \boldsymbol{Z}_m^{(v)}\boldsymbol{H}_m^{(v)}$, so Eq. (3) can be written as follows.

$$\begin{aligned} &\left\| \boldsymbol{X}^{(v)} - \phi\boldsymbol{Z}_i^{(v)}\boldsymbol{H}_i^{(v)} \right\|_F^2 \\ &= tr(\boldsymbol{X}^{(v)T}\boldsymbol{X}^{(v)} - \boldsymbol{X}^{(v)T}\phi\boldsymbol{Z}_i^{(v)}\boldsymbol{H}_i^{(v)} - \boldsymbol{H}_i^{(v)T}\boldsymbol{Z}_i^{(v)T}\phi^T\boldsymbol{X}^{(v)} + \boldsymbol{H}_i^{(v)T}\boldsymbol{Z}_i^{(v)T}\phi^T\phi\boldsymbol{Z}_i^{(v)}\boldsymbol{H}_i^{(v)}) \end{aligned} \quad (4)$$

Take the partial derivative of the above formula with respect to $\boldsymbol{Z}_i^{(v)}$ and set it equal to 0. Since the first term of Eq. (4) is independent of $\boldsymbol{Z}_i^{(v)}$, the first term is not considered in the derivation. The partial derivative of the second term of Eq. (4) is equal to Eq. (5)

$$\frac{\partial tr\left(-\boldsymbol{X}^{(v)T}\phi\boldsymbol{Z}_i^{(v)}\boldsymbol{H}_i^{(v)}\right)}{\partial\left(\boldsymbol{Z}_i^{(v)}\right)} = -\phi^T\boldsymbol{X}^{(v)}\boldsymbol{H}_i^{(v)T} \quad (5)$$

The partial derivative of third term of Eq. (4) is as follows:

$$\frac{\partial tr\left(-\boldsymbol{H}_i^{(v)T}\boldsymbol{Z}_i^{(v)T}\phi^T\boldsymbol{X}^{(v)}\right)}{\partial\left(\boldsymbol{Z}_i^{(v)}\right)} = -\phi^T\boldsymbol{X}^{(v)}\boldsymbol{H}_i^{(v)T} \quad (6)$$

The partial derivative of fourth term of Eq. (4) is as follows:

$$\frac{\partial tr\left(\phi^T\phi \boldsymbol{Z}_i^{(v)}\boldsymbol{H}_i^{(v)}\boldsymbol{H}_i^{(v)T}\boldsymbol{Z}_i^{(v)T}\right)}{\partial\left(\boldsymbol{Z}_i^{(v)}\right)} = 2\phi^T\phi \boldsymbol{Z}_i^{(v)}\boldsymbol{H}_i^{(v)}\boldsymbol{H}_i^{(v)T} \tag{7}$$

Then, we can set the partial derivative to 0,

$$-\phi^T\boldsymbol{X}^{(v)}\boldsymbol{H}_i^{(v)T} - \phi^T\boldsymbol{X}^{(v)}\boldsymbol{H}_i^{(v)T} + 2\phi^T\phi\boldsymbol{Z}_i^{(v)}\boldsymbol{H}_i^{(v)}\boldsymbol{H}_i^{(v)T} = 0 \tag{8}$$

We obtain:

$$\boldsymbol{Z}_i^{(v)} = \boldsymbol{X}\phi^{-1}\boldsymbol{X}^{(v)}(\boldsymbol{H}_i^{(v)})^{-1} \tag{9}$$

For $\boldsymbol{H}_m^{(v)}$, Fix the remaining variables and solve by minimizing Eq. (10), which is as follows.

$$\begin{aligned} &\min_{\boldsymbol{H}_m^{(v)}} \sum_{v=1}^{V}\left(\left\|\boldsymbol{X}^{(v)} - \boldsymbol{Z}_1^{(v)}\boldsymbol{Z}_2^{(v)}\ldots\boldsymbol{Z}_m^{(v)}\boldsymbol{H}_m^{(v)}\right\|_F^2 + \lambda_1\sum_j\left\|\left(\boldsymbol{H}_m^{(v)}\right)_{.j}\right\|_1^2 + \lambda_2\left\|\boldsymbol{H}_m^{(v)} - \boldsymbol{H}\boldsymbol{G}^{(v)}\right\|_F^2\right) \\ &s.t.\boldsymbol{H}_m^{(v)} \geq 0 \end{aligned} \tag{10}$$

Let $\boldsymbol{\Psi} = \boldsymbol{Z}_1^{(v)}\boldsymbol{Z}_2^{(v)}\ldots\boldsymbol{Z}_m^{(v)}$. Since $\boldsymbol{H}_m^{(v)}$ of each omics data is different, the partial derivative is calculated on each omics data. The optimization function can be written as:

$$\begin{aligned} F &= \left\|\boldsymbol{X}^{(v)} - \boldsymbol{\Psi}\boldsymbol{H}_m^{(v)}\right\|_F^2 + \lambda_1\sum_j\left\|\left(\boldsymbol{H}_m^{(v)}\right)_{.j}\right\|_1^2 + \lambda_2\left\|\boldsymbol{H}_m^{(v)} - \boldsymbol{H}\boldsymbol{G}^{(v)}\right\|_F^2 \\ &= tr\left[\boldsymbol{X}^{(v)T}\boldsymbol{X}^{(v)} - \boldsymbol{H}_m^{(v)T}\boldsymbol{\Psi}^T\boldsymbol{X}^{(v)} - \boldsymbol{X}^{(v)T}\boldsymbol{\Psi}\boldsymbol{H}_m^{(v)} + \boldsymbol{H}_m^{(v)T}\boldsymbol{\Psi}^T\boldsymbol{\Psi}\boldsymbol{H}_m^{(v)}\right] \\ &\quad +\lambda_1 tr\left[\boldsymbol{H}_m^{(v)}\boldsymbol{H}_m^{(v)T}\boldsymbol{E}\right] + \lambda_2 tr\left[\boldsymbol{H}_m^{(v)T}\boldsymbol{H}_m^{(v)} - \boldsymbol{G}^{(v)T}\boldsymbol{H}^T\boldsymbol{H}_m^{(v)T} - \boldsymbol{H}_m^{(v)T}\boldsymbol{H}\boldsymbol{G}^{(v)} + \boldsymbol{G}^{(v)T}\boldsymbol{H}^T\boldsymbol{H}\boldsymbol{G}^{(v)}\right] \end{aligned} \tag{11}$$

According to the KKT condition and Lagrangian function, Eq. (11) can be constructed as follows:

$$\mathcal{L} = F - tr\left(\because_m \boldsymbol{H}_m^{(v)T}\right) \tag{12}$$

In Eq. (12), $\because_m$ is a Lagrange multiplier. Take the partial derivative of Eq. (12) as follows:

$$\frac{\partial\mathcal{L}}{\partial\boldsymbol{H}_m^{(v)}} = -2\boldsymbol{\Psi}^T\boldsymbol{X}^{(v)} + 2\boldsymbol{\Psi}^T\boldsymbol{\Psi}\boldsymbol{H}_m^{(v)} + 2\lambda_1 ee^T\boldsymbol{H}_m^{(v)} + 2\lambda_2\left(\boldsymbol{H}_m^{(v)} - \boldsymbol{H}\boldsymbol{G}^{(v)}\right) - \because_m \tag{13}$$

set $\frac{\partial\mathcal{L}}{\partial H_m^{(v)}} = 0$, so the following Eq. (14) can be obtained.

$$-\boldsymbol{\Psi}^T\boldsymbol{X}^{(v)} + \boldsymbol{\Psi}^T\boldsymbol{\Psi}\boldsymbol{H}_m^{(v)} + \lambda_1 ee^T\boldsymbol{H}_m^{(v)} + \lambda_2\left(\boldsymbol{H}_m^{(v)} - \boldsymbol{H}\boldsymbol{G}^{(v)}\right) = \frac{1}{2}\because_m \tag{14}$$

If the matrices are equal, then the elements of the matrices are also equal, so the Eq. (15) can be obtained.

$$
\begin{aligned}
&\left[-\boldsymbol{\Psi}^T\boldsymbol{X}^{(v)}+\boldsymbol{\Psi}^T\boldsymbol{\Psi}\boldsymbol{H}_m^{(v)}+\lambda_1 ee^T\boldsymbol{H}_m^{(v)}+\lambda_2\left(\boldsymbol{H}_m^{(v)}-\boldsymbol{H}\boldsymbol{G}^{(v)}\right)\right]_{ij}=\frac{1}{2}[\because_m]_{ij}\\
&\left(-\boldsymbol{\Psi}^T\boldsymbol{X}^{(v)}+\boldsymbol{\Psi}^T\boldsymbol{\Psi}\boldsymbol{H}_m^{(v)}+\lambda_1 ee^T\boldsymbol{H}_m^{(v)}+\lambda_2\boldsymbol{H}_m^{(v)}-\lambda_2\boldsymbol{H}\boldsymbol{G}^{(v)}\right)_{ij}\left(\boldsymbol{H}_m^{(v)}\right)_{ij}\\
&=(\because_m)_{ij}(\boldsymbol{H}_m^{(v)})_{ij}=0
\end{aligned}
\tag{15}
$$

Only considering $\boldsymbol{H}_m^{(v)}$ and removing the terms irrelevant to $\boldsymbol{H}_m^{(v)}$, Eq. (11) can be written as follows:

$$
\begin{aligned}
F\left(\boldsymbol{H}_m^{(v)}\right)=tr&\left[-\boldsymbol{H}_m^{(v)T}\boldsymbol{\Psi}^T\boldsymbol{X}^{(v)}-\boldsymbol{X}^{(v)T}\boldsymbol{\Psi}\boldsymbol{H}_m^{(v)}+\boldsymbol{H}_m^{(v)T}\boldsymbol{\Psi}^T\boldsymbol{\Psi}\boldsymbol{H}_m^{(v)}\right]\\
&+\lambda_1 tr\left[\boldsymbol{H}_m^{(v)}\boldsymbol{H}_m^{(v)T}\boldsymbol{E}\right]+\lambda_2 tr\left[\boldsymbol{H}_m^{(v)T}\boldsymbol{H}_m^{(v)}-\boldsymbol{G}^{(v)T}\boldsymbol{H}^T\boldsymbol{H}_m^{(v)}-\boldsymbol{H}_m^{(v)T}\boldsymbol{H}\boldsymbol{G}^{(v)}\right]
\end{aligned}
\tag{16}
$$

According to the properties of the trace, Eq. (16) can be written as follows:

$$
\begin{aligned}
F(\mathbf{A})&=tr\left[-\boldsymbol{A}^T\boldsymbol{\Psi}^T\boldsymbol{X}^{(v)}-\boldsymbol{X}^{(v)T}\boldsymbol{\Psi}\boldsymbol{A}+\boldsymbol{A}^T\boldsymbol{\Psi}^T\boldsymbol{\Psi}\boldsymbol{A}\right]+\lambda_1 tr[\boldsymbol{A}\boldsymbol{A}^T\boldsymbol{E}]+\lambda_2 tr\left[\boldsymbol{A}^T\boldsymbol{I}\boldsymbol{A}-\boldsymbol{G}^{(v)T}\boldsymbol{H}^T\boldsymbol{A}-\boldsymbol{A}^T\boldsymbol{H}\boldsymbol{G}^{(v)}\right]\\
&=tr\left[-2\boldsymbol{A}^T\boldsymbol{\Psi}^T\boldsymbol{X}^{(v)}+\boldsymbol{A}^T\boldsymbol{\Psi}^T\boldsymbol{\Psi}\boldsymbol{A}\right]+tr[\boldsymbol{A}\boldsymbol{A}^T\lambda_1\boldsymbol{E}]+tr\left[\boldsymbol{A}^T\lambda_2\boldsymbol{I}\boldsymbol{A}-2\boldsymbol{A}^T\lambda_2\boldsymbol{H}\boldsymbol{G}^{(v)}\right]\\
&=tr\left(-2\boldsymbol{A}^T\boldsymbol{\Psi}^T\boldsymbol{X}^{(v)}\right)+tr(\boldsymbol{A}^T\boldsymbol{\Psi}^T\boldsymbol{\Psi}\boldsymbol{A})+tr(\boldsymbol{A}^T\lambda_1\boldsymbol{E}\boldsymbol{A})+tr(\boldsymbol{A}^T\lambda_2\boldsymbol{I}\boldsymbol{A})+tr\left(-2\boldsymbol{A}^T\lambda_2\boldsymbol{H}\boldsymbol{G}^{(v)}\right)\\
&=tr\left[-2\boldsymbol{A}^T\left(\boldsymbol{\Psi}^T\boldsymbol{X}^{(v)}+\lambda_2\boldsymbol{H}\boldsymbol{G}^{(v)}\right)+\boldsymbol{A}^T(\boldsymbol{\Psi}^T\boldsymbol{\Psi}+\lambda_1\boldsymbol{E}+\lambda_2\boldsymbol{I})\boldsymbol{A}\right]\\
&=tr(-2\boldsymbol{A}^T\boldsymbol{B}+\boldsymbol{A}^T\boldsymbol{C}\boldsymbol{A})\\
&=tr(-2\boldsymbol{A}^T\boldsymbol{B}^++2\boldsymbol{A}^T\boldsymbol{B}^-+\boldsymbol{A}^T\boldsymbol{C}^+\boldsymbol{A}-\boldsymbol{A}^T\boldsymbol{C}^-\boldsymbol{A})
\end{aligned}
\tag{17}
$$

Among them, $\boldsymbol{A}=(\boldsymbol{H}_m^{(v)})$, $\boldsymbol{I}$ is the identity matrix, $\boldsymbol{B}=\boldsymbol{\Psi}^T\boldsymbol{X}^{(v)}+\lambda_2\boldsymbol{H}\boldsymbol{G}^{(v)},\boldsymbol{C}=\boldsymbol{\Psi}^T\boldsymbol{\Psi}+\lambda_1\boldsymbol{E}+\lambda_2\boldsymbol{I}$. The update method of $\boldsymbol{A}$ is as follows Eq. (18), and the specific derivation (Ding et al., 2008) is ignored in this paper.

$$
\boldsymbol{A}_{ik}\leftarrow\boldsymbol{A}_{ik}\sqrt{\frac{\boldsymbol{B}_{ik}^{+}+(\boldsymbol{C}^-\boldsymbol{A})_{ik}}{\boldsymbol{B}_{ik}^{-}+(\boldsymbol{C}^+\boldsymbol{A})_{ik}}}
\tag{18}
$$

For $\boldsymbol{H}_i^{(v)}$ $(i<m)$, fix the remaining variables and solve by minimizing Eq. (19).

$$
\min_{\boldsymbol{H}_m^{(v)},\boldsymbol{H}}\sum_{v=1}^{V}\left\|\boldsymbol{X}^{(v)}-\boldsymbol{Z}_1^{(v)}\boldsymbol{Z}_2^{(v)}\ldots\boldsymbol{Z}_i^{(v)}\boldsymbol{H}_i^{(v)}\right\|_F^2
\tag{19}
$$

Set $\boldsymbol{\Psi}=\boldsymbol{Z}_1^{(v)}\boldsymbol{Z}_2^{(v)}\ldots\boldsymbol{Z}_i^{(v)}$, so the following Eq. (20) can be obtained.

$$
\begin{aligned}
\left\|\boldsymbol{X}^{(v)}-\boldsymbol{Z}_1^{(v)}\boldsymbol{Z}_2^{(v)}\ldots\boldsymbol{Z}_i^{(v)}\boldsymbol{H}_i^{(v)}\right\|_F^2&=\left\|\boldsymbol{X}^{(v)}-\boldsymbol{\Psi}\boldsymbol{H}_i^{(v)}\right\|_F^2\\
&=tr\left[\boldsymbol{X}^{(v)T}\boldsymbol{X}^{(v)}-\boldsymbol{H}_i^{(v)T}\boldsymbol{\Psi}^T\boldsymbol{X}^{(v)}-\boldsymbol{X}^{(v)T}\boldsymbol{\Psi}\boldsymbol{H}_i^{(v)}+\boldsymbol{H}_i^{(v)T}\boldsymbol{\Psi}^T\boldsymbol{\Psi}\boldsymbol{H}_i^{(v)}\right]
\end{aligned}
\tag{20}
$$

Remove the terms in Eq. (20) that are not related to $\boldsymbol{H}_i^{(v)}$ and rewrite it as follows.

$$
F\left(\boldsymbol{H}_i^{(v)}\right)=tr\left[-2\boldsymbol{H}_i^{(v)T}\boldsymbol{\Psi}^T\boldsymbol{X}^{(v)}+\boldsymbol{H}_i^{(v)T}\boldsymbol{\Psi}^T\boldsymbol{\Psi}\boldsymbol{H}_i^{(v)}\right]
\tag{21}
$$

Therefore, the update method of $\boldsymbol{H}_i^{(v)}$ is as follows, and the specific derivation (Ding et al., 2008) is ignored in this paper.

$$
\boldsymbol{H}_{ik}^{(v)}\leftarrow\boldsymbol{H}_{ik}^{(v)}\sqrt{\frac{(\boldsymbol{\Psi}^T\boldsymbol{X}^{(v)})_{ik}^{+}+\left((\boldsymbol{\Psi}^T\boldsymbol{\Psi}^-)\boldsymbol{H}\right)_{ik}}{(\boldsymbol{\Psi}^T\boldsymbol{X}^{(v)})_{ik}^{-}+\left((\boldsymbol{\Psi}^T\boldsymbol{\Psi}^-)\boldsymbol{H}\right)_{ik}}}
\tag{22}
$$

For $\boldsymbol{H}$, Fix the remaining variables and solve by minimizing Eq. (23).

$$\min_{\boldsymbol{H}} \sum_{v=1}^{V} \left\|\boldsymbol{H}_m^{(v)} - \boldsymbol{H}\boldsymbol{G}^{(v)}\right\|_F^2 \tag{23}$$

Eq. (23) can be written as follows:

$$\begin{aligned}&\sum_{v=1}^{V} \left\|\boldsymbol{H}_m^{(v)} - \boldsymbol{H}\boldsymbol{G}^{(v)}\right\|_F^2 \\ &= \sum_{v=1}^{V} tr\left[\boldsymbol{H}_m^{(v)T}\boldsymbol{H}_m^{(v)} - \boldsymbol{G}^{(v)T}\boldsymbol{H}^T\boldsymbol{H}_m^{(v)T} - \boldsymbol{H}_m^{(v)T}\boldsymbol{H}\boldsymbol{G}^{(v)} + \boldsymbol{G}^{(v)T}\boldsymbol{H}^T\boldsymbol{H}\boldsymbol{G}^{(v)}\right]\end{aligned} \tag{24}$$

Remove the terms that are not related to $\boldsymbol{H}$ and set partial derivative to 0, then the following Eq. (25) can be obtained.

$$\begin{gathered}\sum_{v=1}^{V} \left(-\boldsymbol{H}_m^{(v)}\boldsymbol{G}^{(v)T} + \boldsymbol{H}\boldsymbol{G}^{(v)}\boldsymbol{G}^{(v)T}\right) = 0 \\ \boldsymbol{H}\sum_{v=1}^{V} \boldsymbol{G}^{(v)}\boldsymbol{G}^{(v)T} = \sum_{v=1}^{V} \boldsymbol{H}_m^{(v)}\boldsymbol{G}^{(v)T}\end{gathered} \tag{25}$$

So the way to update $\boldsymbol{H}$ is as follows:

$$\boldsymbol{H} = \sum_{v=1}^{V} \boldsymbol{H}_m^{(v)}\boldsymbol{G}^{(v)T}\left(\sum_{v=1}^{V} \boldsymbol{G}^{(v)}\boldsymbol{G}^{(v)T}\right)^{-1} \tag{26}$$

Supplementary Note 2. Deep Matrix Nonlinear Factorization

The optimization goal of the deep matrix nonlinear factorization model is as follows.

$$\begin{aligned}&\min_{\boldsymbol{Z}_i^{(v)},\boldsymbol{H}} \sum_{v=1}^{V} \left(\left\|\boldsymbol{X}^{(v)} - \boldsymbol{Z}_1^{(v)} f\left(\boldsymbol{Z}_2^{(v)} f\left(\dots f\left(\boldsymbol{Z}_m^{(v)}\boldsymbol{H}_m^{(v)}\right)\right)\right)\right\|_F^2 + \lambda_1 \sum_j \left\|\left(\boldsymbol{H}_m^{(v)}\right)_{.j}\right\|_1^2 + \lambda_2 \left\|\boldsymbol{H}_m^{(v)} - \boldsymbol{H}\boldsymbol{G}^{(v)}\right\|_F^2\right) \\ &s.t. \boldsymbol{H}_m^{(v)} \geq 0\end{aligned} \tag{27}$$

To solve the optimization goal of the deep matrix nonlinear factorization model, the gradient of each variable is first solved. Each variable is regarded as the only variable of the objective function, and its partial derivative is taken as the gradient. The variable is updated using the gradient descent method, and the optimal solution is solved by alternately updating the variables.

For $\boldsymbol{H}_m^{(v)}$ $(1 \leq i \leq m)$, fix other variables and the gradient is calculated by taking the partial derivative of equation (27) with respect to $\boldsymbol{H}_m^{(v)}$.

$$\mathcal{L} = \sum_{v=1}^{V} \left(\left\|\boldsymbol{X}^{(v)} - \boldsymbol{Z}_1^{(v)} f\left(\boldsymbol{Z}_2^{(v)} f\left(\dots f\left(\boldsymbol{Z}_m^{(v)}\boldsymbol{H}_m^{(v)}\right)\right)\right)\right\|_F^2 + \lambda_1 \sum_j \left\|\left(\boldsymbol{H}_m^{(v)}\right)_{.j}\right\|_1^2 + \lambda_2 \left\|\boldsymbol{H}_m^{(v)} - \boldsymbol{H}\boldsymbol{G}^{(v)}\right\|_F^2\right) \tag{28}$$

Calculate the gradient of $\boldsymbol{H}_m^{(v)}$ in each omics data separately, as follows:

$$\begin{aligned}\frac{\partial \mathcal{L}}{\partial \boldsymbol{H}_m^{(v)}} &= \frac{\partial\left(\boldsymbol{Z}_m^{(v)}\boldsymbol{H}_m^{(v)}\right)}{\partial \boldsymbol{H}_m^{(v)}}\frac{\partial \mathcal{L}}{\partial\left(\boldsymbol{Z}_m^{(v)}\boldsymbol{H}_m^{(v)}\right)} + 2\lambda_1 ee^T\boldsymbol{H}_m^{(v)} + 2\lambda_2\left(\boldsymbol{H}_m^{(v)} - \boldsymbol{H}\boldsymbol{G}^{(v)}\right)\\ &= \boldsymbol{Z}_{\boldsymbol{m}}^{(\boldsymbol{v})\boldsymbol{T}}\frac{\partial \mathcal{L}}{\partial\left(\boldsymbol{Z}_m^{(v)}\boldsymbol{H}_m^{(v)}\right)} + 2\lambda_1 ee^T\boldsymbol{H}_m^{(v)} + 2\lambda_2\left(\boldsymbol{H}_m^{(v)} - \boldsymbol{H}\boldsymbol{G}^{(v)}\right)\\ &= \mathbf{Z}_m^{(v)T}\frac{\partial \mathcal{L}}{\partial f\left(\boldsymbol{Z}_m^{(v)}\boldsymbol{H}_m^{(v)}\right)}\frac{\partial f\left(\boldsymbol{Z}_m^{(v)}\boldsymbol{H}_m^{(v)}\right)}{\partial\left(\boldsymbol{Z}_m^{(v)}\boldsymbol{H}_m^{(v)}\right)} + 2\lambda_1 ee^T\boldsymbol{H}_m^{(v)} + 2\lambda_2\left(\boldsymbol{H}_m^{(v)} - \boldsymbol{H}\boldsymbol{G}^{(v)}\right)\\ &= \mathbf{Z}_m^{(v)T}\left[\frac{\partial \mathcal{L}}{\partial \boldsymbol{H}_{m-1}^{(v)}} \odot \nabla f\left(\boldsymbol{Z}_m^{(v)}\boldsymbol{H}_m^{(v)}\right)\right] + 2\lambda_1 ee^T\boldsymbol{H}_m^{(v)} + 2\lambda_2\left(\boldsymbol{H}_m^{(v)} - \boldsymbol{H}\boldsymbol{G}^{(v)}\right)\end{aligned} \tag{29}$$

Among them, $\frac{\partial \mathcal{L}}{\partial \boldsymbol{H}_i^{(v)}}$ is the partial derivative of Eq. (30) with respect to $\boldsymbol{H}_i^{(v)}$, the mathematical expression is as follows:

$$\begin{aligned}\frac{\partial \mathcal{L}}{\partial \boldsymbol{H}_i^{(v)}} &= \frac{\partial\left(\boldsymbol{Z}_i^{(v)}\boldsymbol{H}_i^{(v)}\right)}{\partial \boldsymbol{H}_i^{(v)}}\frac{\partial \mathcal{L}}{\partial\left(\boldsymbol{Z}_i^{(v)}\boldsymbol{H}_i^{(v)}\right)}\\ &= \boldsymbol{Z}_i^{(v)T}\frac{\partial \mathcal{L}}{\partial\left(\boldsymbol{Z}_i^{(v)}\boldsymbol{H}_i^{(v)}\right)}\\ &= \boldsymbol{Z}_i^{(v)T}\frac{\partial \mathcal{L}}{\partial f\left(\boldsymbol{Z}_i^{(v)}\boldsymbol{H}_i^{(v)}\right)}\frac{\partial f\left(\boldsymbol{Z}_i^{(v)}\boldsymbol{H}_i^{(v)}\right)}{\partial\left(\boldsymbol{Z}_i^{(v)}\boldsymbol{H}_i^{(v)}\right)}\\ &= \boldsymbol{Z}_i^{(v)T}\left[\frac{\partial \mathcal{L}}{\partial \boldsymbol{H}_{i-1}^{(v)}} \odot \nabla f\left(\boldsymbol{Z}_i^{(v)}\boldsymbol{H}_i^{(v)}\right)\right]\end{aligned} \tag{30}$$

When $i = 2$, the gradient of $\boldsymbol{H}_1^{(v)}$ is converted into a linear model, and Eq. (28) can be written as:

$$\begin{aligned}\mathcal{L} &= \sum_{v=1}^{V}\left\|\boldsymbol{X}^{(v)} - \boldsymbol{Z}_1^{(v)}\boldsymbol{H}_1^{(v)}\right\|_F^2\\ &= tr\left(\boldsymbol{X}^{(v)T}\boldsymbol{X}^{(v)} - \boldsymbol{H}_1^{(v)T}\boldsymbol{Z}_1^{(v)}\boldsymbol{X}^{(v)} - \boldsymbol{X}^{(v)T}\boldsymbol{Z}_1^{(v)}\boldsymbol{H}_1^{(v)} + \boldsymbol{H}_1^{(v)T}\boldsymbol{Z}_1^{(v)^T}\boldsymbol{Z}_1^{(v)}\boldsymbol{H}_1^{(v)}\right)\end{aligned} \tag{31}$$

Eq. (32) for calculating the partial derivative of Eq. (31) with respect to $\boldsymbol{H}_1^{(v)}$ can be written as:

$$\begin{aligned}\frac{\partial \mathcal{L}}{\partial \boldsymbol{H}_1^{(v)}} &= -2\boldsymbol{Z}_1^{(v)^T}\boldsymbol{X}^{(v)} + 2\boldsymbol{Z}_1^{(v)^T}\boldsymbol{Z}_1^{(v)}\boldsymbol{H}_1^{(v)}\\ &= \boldsymbol{Z}_{\boldsymbol{1}}^{(\boldsymbol{v})\boldsymbol{T}}\left(2\boldsymbol{Z}_{\boldsymbol{1}}^{(v)}\boldsymbol{H}_1^{(v)} - 2\boldsymbol{X}^{(v)}\right)\end{aligned} \tag{32}$$

According to the chain rule, as long as the gradient of $\boldsymbol{H}_1^{(v)}$ is calculated, the gradient of $\boldsymbol{H}_i^{(v)}$ $(1 < i \leq m)$ can be calculated. Set the step size to α, and the update method is as follows:

$$\begin{aligned}\boldsymbol{H}_1^{(v)} &= \boldsymbol{H}_1^{(v)} - \alpha\frac{\partial \mathcal{L}}{\partial \boldsymbol{H}_1^{(v)}}\\ \boldsymbol{H}_2^{(v)} &= \boldsymbol{H}_2^{(v)} - \alpha\frac{\partial \mathcal{L}}{\partial \boldsymbol{H}_2^{(v)}}\\ &\vdots\\ \boldsymbol{H}_i^{(v)} &= \boldsymbol{H}_i^{(v)} - \alpha\frac{\partial \mathcal{L}}{\partial \boldsymbol{H}_i^{(v)}}\end{aligned} \tag{33}$$

For $\boldsymbol{Z}_i^{(v)}$ $(1 \le i \le m)$, fix other variables and calculate the gradient by taking the partial derivative of Eq. (28) with respect to $\boldsymbol{Z}_i^{(v)}$.

$$\mathcal{L} = \sum_{v=1}^{V} \left(\left\| \boldsymbol{X}^{(v)} - \boldsymbol{Z}_1^{(v)} f\left(\boldsymbol{Z}_2^{(v)} f\left(\dots f\left(\boldsymbol{Z}_m^{(v)} \boldsymbol{H}_m^{(v)} \right) \right) \right) \right\|_F^2 \right) \tag{34}$$

Because each omics data is separate, calculations are performed on each omics. The calculation formula is as follows.

$$\begin{aligned} \frac{\partial \mathcal{L}}{\partial \boldsymbol{Z}_i^{(v)}} &= \frac{\partial \mathcal{L}}{\partial \left(\boldsymbol{Z}_i^{(v)} \boldsymbol{H}_i^{(v)} \right)} \boldsymbol{H}_i^{(v)T} \\ &= \left[\frac{\partial \mathcal{L}}{\partial \boldsymbol{H}_{i-1}^{(v)}} \odot \nabla f\left(\boldsymbol{Z}_i^{(v)} \boldsymbol{H}_i^{(v)} \right) \right] \boldsymbol{H}_i^{(v)T} \end{aligned} \tag{35}$$

Among them, when $i = 2$, the calculation of the gradient of $\boldsymbol{Z}_1^{(v)}$ is converted into a linear model, and Eq. (28) can be written as:

$$\begin{aligned} \mathcal{L} &= \sum_{v=1}^{V} \left\| \boldsymbol{X}^{(v)} - \boldsymbol{Z}_1^{(v)} \boldsymbol{H}_1^{(v)} \right\|_F^2 \\ &= tr\left(\boldsymbol{X}^{(v)T} \boldsymbol{X}^{(v)} - \boldsymbol{H}_1^{(v)T} \boldsymbol{Z}_1^{(v)} \boldsymbol{X}^{(v)} - \boldsymbol{X}^{(v)T} \boldsymbol{Z}_1^{(v)} \boldsymbol{H}_1^{(v)} + \boldsymbol{H}_1^{(v)T} \boldsymbol{Z}_1^{(v)T} \boldsymbol{Z}_1^{(v)} \boldsymbol{H}_1^{(v)} \right) \end{aligned} \tag{36}$$

So, the partial derivative of Eq. (36) with respect to $\boldsymbol{Z}_1^{(v)}$ can be written as:

$$\frac{\partial \mathcal{L}}{\partial \boldsymbol{Z}_1^{(v)}} = 2\left(\boldsymbol{Z}_1^{(v)} \boldsymbol{H}_1^{(v)} - \boldsymbol{X}^{(v)} \right) \boldsymbol{H}_1^{(v)T} \tag{37}$$

According to the chain rule, as long as the gradient of $\boldsymbol{Z}_1^{(v)}$ is calculated, the gradient of the subsequent $\boldsymbol{Z}_i^{(v)}$ $(1 \le i \le m)$ can be known. Set the step size to α, and the update method is as follows

$$\begin{aligned} \boldsymbol{Z}_1^{(v)} &= \boldsymbol{Z}_1^{(v)} - \alpha \frac{\partial \mathcal{L}}{\partial \boldsymbol{Z}_1^{(v)}} \\ \boldsymbol{Z}_2^{(v)} &= \boldsymbol{Z}_2^{(v)} - \alpha \frac{\partial \mathcal{L}}{\partial \boldsymbol{Z}_2^{(v)}} \\ &\vdots \\ \boldsymbol{Z}_i^{(v)} &= \boldsymbol{Z}_i^{(v)} - \alpha \frac{\partial \mathcal{L}}{\partial \boldsymbol{Z}_i^{(v)}} \end{aligned} \tag{38}$$

For $\boldsymbol{H}$, fix other variables and calculate the gradient by taking the partial derivative of Eq. (28) with respect to $\boldsymbol{H}$.

$$\begin{aligned} \mathcal{L} &= \sum_{v=1}^{V} \lambda_2 \left\| \boldsymbol{H}_m^{(v)} - \boldsymbol{H}\boldsymbol{G}^{(v)} \right\|_F^2 \\ &= \lambda_2 \sum_{v=1}^{V} \left(tr\left(\boldsymbol{H}_m^{(v)^T} \boldsymbol{H}_m^{(v)} - 2\boldsymbol{H}_m^{(v)^T} \boldsymbol{H}\boldsymbol{G}^{(v)} + \boldsymbol{G}^{(v)^T} \boldsymbol{H}^T \boldsymbol{H}\boldsymbol{G}^{(v)} \right) \right) \end{aligned} \tag{39}$$

Remove the terms in Eq. (39) that are not related to $\boldsymbol{H}$ and get the following formula.

$$\mathcal{L} = \lambda_2 \sum_{v=1}^{V} \left(tr\left(-2\boldsymbol{H}_m^{(v)^T} \boldsymbol{H}\boldsymbol{G}^{(v)} + \boldsymbol{G}^{(v)^T} \boldsymbol{H}^T \boldsymbol{H}\boldsymbol{G}^{(v)} \right) \right) \tag{40}$$

Find the partial derivative of Eq. (40) to $\boldsymbol{H}$, the mathematical expression is as follows:

$$\frac{\partial \mathcal{L}}{\partial \boldsymbol{H}} = \partial \lambda_2 \sum_{v=1}^{V} \left(-2\boldsymbol{H}_m^{(v)^T} \boldsymbol{G}^{(v)} + \boldsymbol{H}\boldsymbol{G}^{(v)} \boldsymbol{G}^{(v)^T}\right) \tag{41}$$

Get the gradient of $\boldsymbol{H}$, set the step size to α, and update it as follows.

$$\boldsymbol{H} = \boldsymbol{H} - \alpha \frac{\partial \mathcal{L}}{\partial \boldsymbol{H}} \tag{42}$$

Supplementary Table 1. Table of Dataset Dimensions and Sample Size

Dataset	AML	BIC	COAD	GBM	HNSC	KICH	LIHC	LUSC	OV	SKCM	SARC	METABRIC
Sample Size	170	624	220	274	508	66	367	341	287	450	257	1904

Feature Dimensions:

	AML	BIC	COAD	GBM	HNSC	KICH	LIHC	LUSC	OV	SKCM	SARC
mRNA	2000	2000	2000	2000	2000	2000	2000	2000	2000	2000	2000
methylation	2000	2000	2000	2000	2000	2000	2000	2000	2000	2000	2000
miRNA	558	885	613	534	1000	1000	852	881	616	901	838

	METABRIC
mRNA	2000
CNV	2000

Supplementary Note 3. Biological Interpretability Validation

In order to verify whether the cancer subtypes identified by the MLMF algorithm are biologically meaningful and well interpretable, we performed GO enrichment analysis on the experimental data results. Here we take the enrichment analysis results of the MLMF_Linear algorithm in breast invasive carcinoma (BIC) as an example to illustrate the biological significance of the identified subtypes. As one of the most common malignant tumors in women, BIC has a relatively mature molecular classification system and is an ideal model for verifying the interpretability of subtype analysis algorithms. Through GO enrichment analysis, we can show the effectiveness of the MLMF algorithm in identifying cancer subtypes with real biological basis.

As can be seen from Supplementary Fig. 1, GO enrichment analysis showed that the MLMF_Linear algorithm successfully identified biological pathways of great significance in breast cancer. First, the method accurately identified cell proliferation and cell cycle-related pathways, including key processes such as mitotic nuclear division regulation, mitotic cell cycle, and mitotic nuclear division, which is fully consistent with the known characteristics of breast cancer heterogeneity. This finding is consistent with the pioneering studies of Sørlie et al. and Perou et al.,

which showed that breast cancer subtypes can be effectively distinguished based on the expression patterns of proliferation-related genes, especially Basal-like and Her2-enriched subtypes usually show higher proliferation activity. Second, we observed significant enrichment of extracellular matrix remodeling pathways, which play a key role in breast cancer invasion and metastasis, as demonstrated by Place et al. In addition, MLMF_Linear also identified ion transport regulation pathways, especially potassium ion transmembrane transport regulation, which is consistent with the study of Pardo et al., which demonstrated that potassium channel dysregulation is closely related to breast cancer proliferation, migration, and apoptosis resistance. From a statistical point of view, all enriched GO entries showed significance (p.adjust < 0.05), and the number of enriched genes was moderate (4-10 genes), and the gene ratio was in the range of 0.03-0.10, indicating that the enrichment was both reasonable and not excessive, fully verifying the biological interpretability and reliability of the MLMF_Linear method in breast cancer subtype identification.

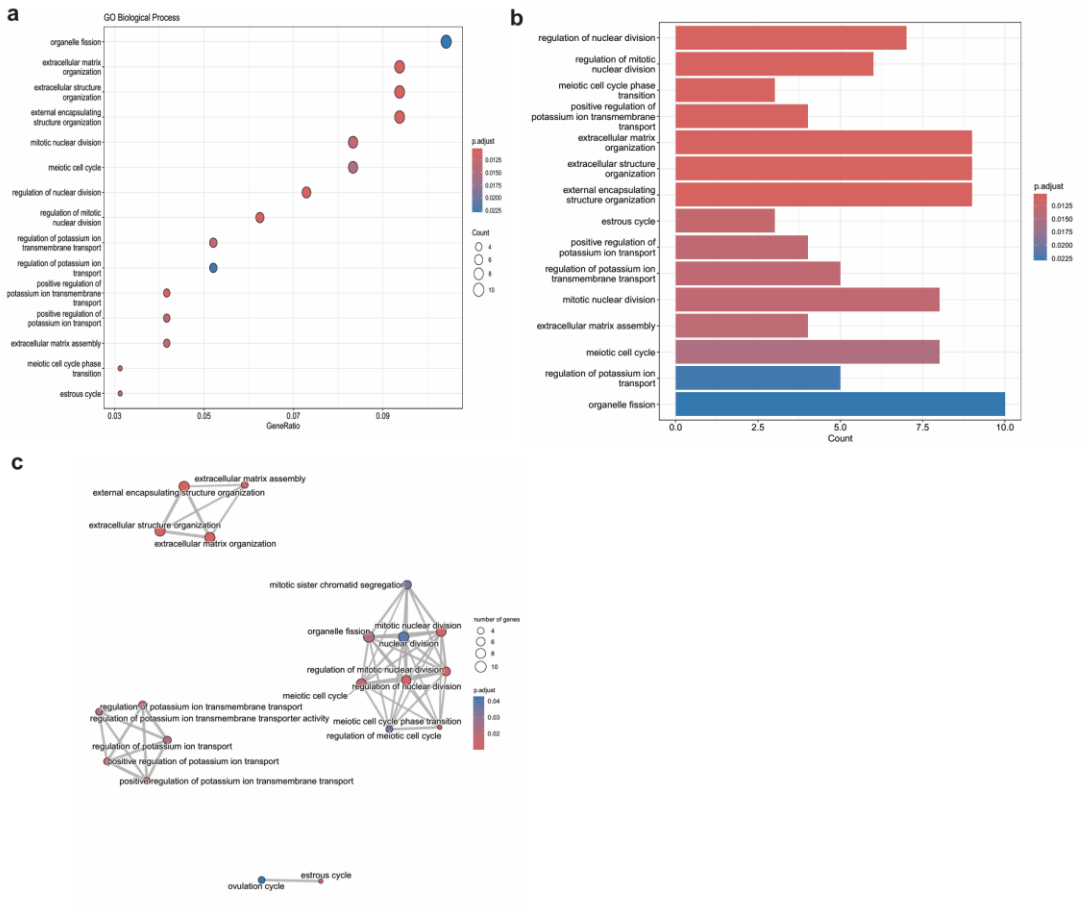

Supplementary Fig. 1. GO Biological Process Enrichment Analysis of BIC Subtypes Identified by MLMF_Linear Algorithm

GO enrichment analysis reveals significant biological processes associated with MLMF_Linear-identified BIC subtypes. (a) Dotplot showing enriched GO terms ranked by gene ratio with p-value significance (color scale). (b) Bar plot displaying count of enriched genes for each GO term with statistical significance (red-blue color scale indicates p.adjust values). (c) Gene concept network illustrating interconnections between enriched GO terms and

associated genes, with node size representing gene count and edge representing gene-pathway associations. Key enriched processes include cell proliferation, extracellular matrix organization, and potassium ion transport regulation, highlighting the biological meaningfulness of the identified subtypes.

Supplementary Fig.2

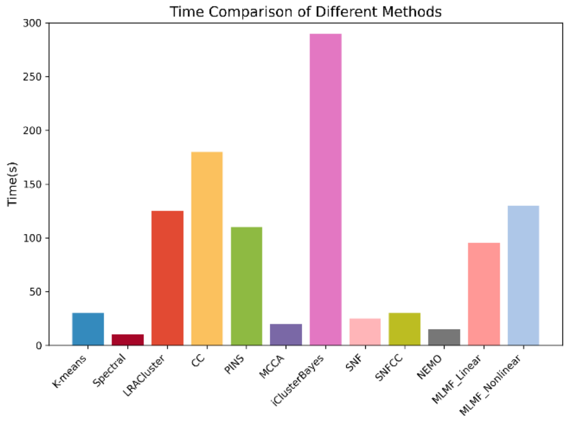

Comparison of the running time of the MLMF_Linear and MLMF_Nonlinear algorithm on the BIC dataset with other algorithms. The Y-axis is time and the X-axis is the algorithm name.

References

Sørlie, T., Perou, C. M., Tibshirani, R., Aas, T., Geisler, S., Johnsen, H., ... & Børresen-Dale, A. L. (2001). Gene expression patterns of breast carcinomas distinguish tumor subclasses with clinical implications. *Proceedings of the National Academy of Sciences*, *98*(19), 10869-10874.

Perou, C. M., Sørlie, T., Eisen, M. B., Van De Rijn, M., Jeffrey, S. S., Rees, C. A., ... & Botstein, D. (2000). Molecular portraits of human breast tumours. *nature*, *406*(6797), 747-752.

Place, A. E., Jin Huh, S., & Polyak, K. (2011). The microenvironment in breast cancer progression: biology and implications for treatment. *Breast cancer research*, *13*, 1-11.

Pardo, L. A., & Stühmer, W. (2014). The roles of K+ channels in cancer. *Nature Reviews Cancer*, *14*(1), 39-48.
